# The Achievement Motive in the Brain: BOLD Responses to Pictures of Challenging Activities Predicted by Implicit Versus Explicit Achievement Motives

**DOI:** 10.3389/fpsyg.2022.845910

**Published:** 2022-07-01

**Authors:** Markus Quirin, Alexander Loktyushin, Ekkehard Küstermann, Julius Kuhl

**Affiliations:** ^1^Department of Sport and Health Sciences, Technical University of Munich, Munich, Germany; ^2^Department of Psychology, PFH Private University of Applied Sciences, Göttingen, Germany; ^3^Department of Empirical Inference, Max-Plank Institute for Intelligent Systems, Tübingen, Germany; ^4^Department of Neuropsychology and Behavioral Neurobiology, University of Bremen, Bremen, Germany; ^5^Institute of Psychology, Osnabrück University, Osnabrück, Germany

**Keywords:** achievement motive, fMRI, implicit vs. explicit motives, operant motives test, motivational neuroscience

## Abstract

The achievement motive refers to a preference for mastering challenges and competing with some standard of excellence. Along with affiliation and power motives, the achievement motive is typically considered to occur on the level of implicit versus explicit representations. Specifically, whereas implicit motives involve pictorial, emotional goal representations and facilitate corresponding action effortlessly, explicit motives involve propositional (“verbalized”) goal representations but need some effort to translate into action (McClelland et al., 1989). We used functional magnetic resonance imaging (fMRI) to investigate whether and to which degree the implicit and explicit achievement motives differentially predict blood-oxygen-level-dependent (BOLD) responses to pictures of individuals engaging in challenging activities. Whereas the implicit AM predicted activity in areas associated with emotion (orbitofrontal cortex) and visual processing (right dorsolateral prefrontal cortex, premotor and occipital cortices), the explicit AM predicted activity in areas associated with cognitive self-control or verbal goal processing (dorsal anterior cingulate cortex, left dorsolateral prefrontal cortex). The findings support the commonly assumed distinction between implicit and explicit motives with neuronal data. They also suggest that explicit motives require cognitive self-control to overcome potential lacks of motivation.

## Introduction

Motives are conceived of as relatively stable individual differences in the preference for approaching certain classes of incentives, such as those related to achievement, power, or affiliation. Their investigation has steadily been a key issue in personality and social psychology since more than half a century (Murray, 1938; McClelland et al., 1953). Because motives specify which situational cues (e.g., achievement-related cues) constitute potential incentives for an individual, they play an important role in explaining human behavior.

Throughout the last decades motives such as for achievement, power or affiliation have either been measured implicitly, such as by researchers interpreting fantasy stories spontaneously generated by participants in response to ambiguous pictures (Murray, 1943), or explicitly, that is, by self-report questionnaires. These two forms of motives are typically uncorrelated (McClelland et al., 1989; Brunstein and Heckhausen, 2018; but see Thrash et al., 2007) and predict mental, behavioral and physiological processes differentially (e.g., McClelland et al., 1989). Therefore, it has thus been postulated that implicit and explicit motives are supported by different psychological systems that might correspond to different networks within the brain (McClelland et al., 1989).

In the present study we focused on the achievement motive (AM) to distinguish neural mechanisms of the two motive systems. Using functional magnetic resonance imaging (fMRI), we investigated whether implicit versus explicit AMs predict brain activity during the presentation of achievement-related stimuli. Finding neural responses to achievement contexts varying in intensity with AMs would add to our knowledge about how the brain functions differentially for individuals with high versus low levels of the AM and would not least allow inferences for neural mechanisms underlying achievement motivation, or human motivation in general. Moreover, finding neural network activation differentially predicted by implicit and explicit AMs would provide support for dual motives theory (McClelland et al., 1989). In the following sections, we will first discuss differences between implicit and explicit motives in general and potential neural correlates, and subsequently focus on specific issues concerning the AM.

### Implicit Versus Explicit Motivational Systems

McClelland et al. (1989) postulated that two different systems underlie implicit versus explicit motives (see also Schultheiss and Brunstein, 2010). The implicit motivational system reflects automatic, often unconscious, affective preferences for a certain class of stimuli and situations. This means that this class of stimuli is of high value for the individual. For example, individuals with high levels of implicit AM typically spontaneously react in a positive way to situations in which they can put their ability to the test. Because the implicit motivation system involves spontaneous and affectively positive reactions to attainable motive-relevant stimuli, behavior can be initiated with little effort and without the necessity of rigorous self-control (e.g., Ainslie, 2021; Quirin et al., 2021). Compatibly, research has demonstrated that implicit motives typically predict spontaneous rather than controlled or socially desired behavior (McClelland et al., 1989).

By contrast, explicit motives refer to individuals’ conscious beliefs and self-reports about themselves, that is, whether they like to put their ability to the test (achievement-related), whether they like to have influence on others (power-related), or whether they like to have close relationships with others (affiliation-related). These beliefs are strongly formed by personal ideals, social norms, expectancies of relevant others, and external rewards such as social acceptance, praise or money. In contrast to implicit motives, self-attitudes (which are predominantly construed on the basis of these external sources) lack inherent and intrinsic positive reactions to motive relevant situations. Therefore, behaviors associated with a particular motive (e.g., putting one’s ability to the test) require cognitive self-control in order to overcome lacks of intrinsic motivation, especially in the absence of extrinsic incentives. Self-control is often implemented or sustained *via* inner verbal self-instructions that aim at the pursuit of goals represented in a propositional format (Kuhl, 2000; Quirin et al., 2021). These self-instructions induce a feeling of burden (“I should” rather than “I want”) and counteract the experience of flow (see Csikszentmihalyi, 1990) –a state closely associated with implicit, intrinsic motivation (e.g., Deci and Ryan, 2008).

In contrast to implicit motives, which are considered to form in early, prelinguistic childhood and to largely maintain throughout a person’s life, explicit motives are less stable and are based on beliefs and attitudes about the personal importance of the respective motive rather than on automatic affective-motivational preferences (McClelland, 1985). Because implicit and explicit motives are considered to develop in an early, prelinguistic versus later, linguistic period of life, respectively, implicit motives are considered to be coded in a pictorial format whereas explicit motives are considered to be coded in a conceptual, propositional format (Atkinson and McClelland, 1948; Schultheiss and Brunstein, 1999, 2010; Langens, 2003).

Given the numerous findings confirming differential predictions for implicit versus explicit motives, McClelland et al. (1989) already speculated that different brain mechanisms may underlie the ways the two motive types affect behavior. To date, however, little empirical evidence supports this notion. Indirect evidence comes from research on implicit power and affiliation motives, which appear to be associated with sex hormones (for an overview, see Schultheiss and Wirth, 2008). Specifically, the implicit power motive (Winter, 1973) has been found to predict increased levels of testosterone, whereas the implicit affiliation motive has been found to predict increased levels of progesterone - hormones the secretion of which underlies activity of brain regions typically not associated with conscious reflection such as the hypothalamus. This is compatible with findings demonstrating absent relationships between explicit motives and those hormones. One study revealed neural correlates of the implicit power motive by demonstrating that this motive moderates neural reactions to angry faces (Schultheiss et al., 2008). Specifically, and in line with the expectation that implicit motives typically predict spontaneous rather than controlled reactions, these authors found activity for individuals with a high implicit power motive in regions associated with the emotional valuation of stimuli and preferences such as the orbitofrontal cortex (OFC). However, in this study the implicit motive was not contrasted with the explicit motive. Whereas these studies generally support the validity of McClelland’s dual-systems model of motives and suggest that linking brain responses to motives is meaningful, investigating links between brain activity and implicit versus explicit AMs is still due.

### The Achievement Motive

Since Achievement Motivation theory originated more than 50 years ago (e.g., McClelland et al., 1953), extensive research has been conducted (for an overview, see Brunstein and Heckhausen, 2018). The AM has been shown to be a relatively stable personality trait as inferable from long-term predictions of life outcome variables (e.g., McClelland and Boyatzis, 1982; McClelland and Franz, 1992; Apers et al., 2019). Individuals with a high AM typically have the ambition to improve their abilities and performances, and thus the tendency to approach challenging tasks (McClelland et al., 1953; Elliot and Thrash, 2001; Schüler et al., 2010, for a study using a semi-implicit measure of achievement).

Empirical findings on life quality point to the relevance of distinguishing between implicit and explicit AMs. For example, McClelland and Franz (1992) found that the implicit AM predicted income and job success 10 years later—a finding that was not replicated for explicit motive measures. In addition, chronic discrepancies between implicit and explicit AMs predicted well-being decrements (Brunstein et al., 1998; Kehr, 2004; Baumann et al., 2005; Langens, 2007; Langan-Fox et al., 2009; see Hofer et al., 2006b, for the affiliation motive) or even psychosomatic complaints (Baumann et al., 2005).

Individuals with a high explicit motive engage in difficult tasks provided those tasks are introduced explicitly (McClelland et al., 1989; Spangler, 1992). However, because the explicit AM does not support spontaneous achievement-related behavior as it is the case for implicit motives, individuals with high levels of explicit and low levels of implicit AM should require high amounts of self-control and *effortful* volitional processes (“strong willpower”; Quirin et al., 2021). These processes should help them overcome the *conflict* between engaging in tasks that are not pleasant in themselves and attaining the rewarding goal (Kehr, 2004; Baumann et al., 2005). Because the task itself is effortful and not inherently rewarding, the explicit AM typically predicts actions that are subject to social evaluation (public behavior) and that are controlled rather than spontaneously initiated (McClelland et al., 1989). Although effortful self-control can be assumed to play a role in explicit motives in general, it can be considered particularly important in the context of explicit achievement because achievement-related actions may in general require higher levels of effort than actions related to power or affiliation.

Still, it needs to be noted that the stimulation of the implicit or explicit AM by an incentive (e.g., a picture of a challenging task) does not immediately translate into manifest, momentary achievement motivation (in a narrow sense) and related neural activation. This is because achievement motivation (and motivation in general) requires the presence of both an incentive and the expectancy to eventually attain it (Beckmann and Heckhausen, 2018).

### Present Research and Hypotheses

The present study was conducted to pilot the neural correlates of reactions to achievement-related stimuli and the moderating roles of implicit versus explicit AMs. To stimulate the AM, we instructed participants to view pictures of protagonists engaging in achievement-related (challenging) activities, and to identify with these protagonists. Differences in BOLD responses to achievement-related pictures (as contrasted with control pictures) were analyzed as a main effect, and regressed on individual differences in the implicit versus explicit AMs in separate analyses.

For the main-effect contrast of achievement versus control pictures we expected to find activation in areas typically related to motivation such as the nucleus accumbens and the OFC. However, such an activation may be moderate because individuals strongly differ in the strength of AMs. In this vein, we expected that the implicit AM predicts activity in regions associated with value and salience representation and related positive emotions (e.g., Phan et al., 2004). such as the OFC (e.g., Kringelbach, 2005) or the insula (Craig, 2002). Because implicit motives are considered to be linked to pictorial rather than verbal goal representations, we additionally expected that motive-relevant situations activate the right DLPFC, which is associated with coding goals in a pictorial format (e.g., Floel et al., 2004). By contrast, the explicit AM should predict activity in regions associated with the overcoming of conflicts such as the Anterior Cingulate Cortex (ACC; e.g., Botvinick et al., 2001; Shackman et al., 2011) as well as in regions associated with self-control and propositional goal representations (“inner verbal self-instructions”), such as the left dorsolateral prefrontal cortex (DLPFC; e.g., Devlin et al., 2003; Floel et al., 2004; Köhler et al., 2004; Kaller et al., 2011).

## Materials and Methods

### Sample

Fifteen students (9 female) from the University of Osnabrück, aged 20–29 (*M* = 25.0, *SD* = 4.2), were recruited *via* placards and received either 30 Euro (about $37.5) or course credit in return for their participation. All were right-handed and had normal, corrected to normal or nearly normal vision. Participants were informed in detail about the fMRI technique, corresponding risks and necessary security measures, and gave written consent to participate. They were also informed that they can abort the experiment at any time when feeling uncomfortable.

### Procedure

First, participants filled in a set of questionnaires and personality tests. Next, but still prior to the MR session participants viewed drawings one by one and in a random order, each accompanied by a short story of two or three sentences describing the situation in the picture. These stories helped standardizing the achievement-related or, for the control pictures, neutral meaning of the situations depicted by the pictures. Participants were instructed to internalize the meaning of the pictures given by the story (cf. Kuhl and Kazén, 2008, for a similar procedure). Next, to enhance the learning process, the pictures were presented solely and participants had to judge whether they remembered the content of the corresponding story or not. If not, that particular picture was repeatedly presented along with the story until the participant indicated to remember the story.

Subsequently, participants were placed into the scanner and were given two computer mize into their left and right hands, respectively. Trials consisted of successive presentations of a blank screen (500 ms), a picture in the center of the screen of either 500 or 3,000 ms duration,^1^ and a red or a blue asterisk in the center of the screen. Participants were instructed to view the pictures and to respond to the asterisk by clicking with their left or right index finger depending on asterisk color. Assignment of key side and color was counterbalanced between subjects. Twenty practice trials were run prior to the actual experiment.

In the experimental phase, 8 blocks of achievement pictures were mixed with 16 blocks of control pictures, with 2 different block orders counterbalanced across participants. Each block consisted of 10 trials with 5 pictures from the same condition being presented twice and in a pseudo-randomized order (no picture in tandem) that was kept constant for all participants. Half of the blocks consisted of mirror-inverted stimulus presentations. Blocks were separated by a 5 s relaxation phase. Stimuli were projected on a screen behind the scanner with a video projector. Participants were able to see the screen *via* a mirror in their head-coil that was positioned for optimal sight before each scan.

To check whether the target pictures aroused achievement-related themes, after being scanned, participants provided Likert ratings from *not at all* (0) to *completely* (9) on the extent to which each picture shares some meaning with the adjectives *concentrated*, *highly efficient* referring to achievement motivation. Because achievement and power motivation sometimes are difficult to distinguish because they share an (outcome-oriented) agency component (Brunstein, 2001; Alsleben and Kuhl, 2010), we wanted to assure that the stimuli are related to achievement rather than to power motivation. To do so, we additionally asked participants to provide ratings for the power-related adjectives *dominant* and *superior*. Ratings were later averaged to obtain one achievement and one power score, respectively. Ratings from the first two participants were missing because of technical issues.

### Stimuli

**FIGURE 1:**
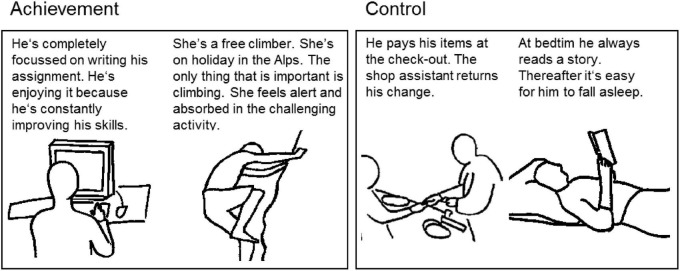
Sample of stimuli related to achievement **(left)** versus control **(right)**.

In all phases of the experiment described before (except for personality assessment), we used E-Prime version 1.1 (Psychology Software Tools Inc.; cf. Schneider et al., 2002) for stimulus presentation and response logging. Experimental stimuli consisted of 5 schematic black drawings depicted against a gray background (rgb = citation{191, 191, 191}) that represented situations in which a target person lives up his or her AM (e.g., a free climber). By contrast, 5 control pictures showed everyday situations involving either non-personal interactions (e.g., paying at the checkout counter) or no interactions at all (e.g., waiting at a bus stop). Figure 1 depicts a sample of the drawings used in the experiment along with their vignettes. Previous research has already successfully used a small number of motive specific, schematic drawings combined with brief vignettes which participants were asked to memorize, in order to stimulate motives other than the AM (Kuhl and Kazén, 2008). Here, we followed this approach to arouse the AM. We did not judge suitable to use a larger number of pictures for our purposes since this would have extended the learning phase well beyond a suitable scope and would have additionally taxed participants’ memory.

To achieve natural body positions, gestures, and proportions, pictures were drawn following the outlines of purpose-made photographs. The number of non-target persons depicted in the pictures were matched between experimental and control groups. The drawings were kept as simple as possible to minimize complexity of the pictures thereby controlling for physical aspects between the single pictures such as color and luminance, and not to distract individuals from irrelevant aspects of the drawings. Based on continuous discussions among five experts from the area of motive psychology and additional discussions in two lab meetings, pictures from the initial set were drawn, redrawn, or substituted in order to obtain pictures that are most unambiguous with respect to their motivational content.

### Psychological Assessment

We applied the *Personal Values Questionnaire* (PVQ; McClelland, 1991) for the assessment of the explicit AM and the *Operant Motive Test* (OMT; Kuhl et al., 2003; Scheffer et al., 2003) for the assessment of the implicit AM. In the PVQ participants are instructed to “rate how important each item is to you” on a 6-point Likert scale from (0) *not important* to (5) *extremely important*. Typical achievement-related items (out of a total of twelve) are *opportunities to take on more difficult and challenging goals and responsibilities* or *personally doing things better than they have been done before*. Cronbach’s Alpha of the scale was 0.89. The OMT is a variant of the Thematic Apperception Test (Morgan and Murray, 1935), in which participants are asked to imagine stories to a given picture. Unlike the TAT, the OMT asks participants to answer in note form four questions about what is going on in a picture rather than to write down complete stories. Moreover, the OMT uses a set of 15 pictures, which is about three times as much as typically used in TAT research forms (Schultheiss and Brunstein, 2001). Each picture story classified as being achievement-related adds one point to the AM scale ranging from 0 to 15. Subscales of the OMT showed adequate test properties (Runge et al., 2016) and predicted behavioral and physiological responses in more than 50 peer-reviewed articles (e.g., Scheffer et al., 2003; Baumann et al., 2005; Hofer et al., 2006a,b; Quirin et al., 2009, 2013). In the present study, classification was performed by two professional OMT raters who showed an inter-rater consistency of 0.92.

### MR Data Acquisition

Data were acquired by a 3 Tesla head scanner (Siemens Allegra system, Siemens, Erlangen, Germany) at the University of Bremen with a 40mT gradient coil and a circular polarized send and receive head coil. Participants wore ear plugs for their protection, and their head was fixated by rolls of foam to avoid head motion artifacts. In case subjects felt unwell during the scan or wanted to abort the experiment for any other reason, they had the possibility to press an emergency ball which was lying within reach during the entire scan.

A preliminary scan served to localize the head position, where the brain was displayed in three orthogonal slices, such that it was possible to adjust slices in a way that the frontal cortex was fully included. Subsequent functional scans were acquired in 36 slices with a spatial resolution of 3 × 3 mm and a slice thickness of 3 mm. These slices were acquired in an interleaved order from bottom to top. They were positioned obliquely to the line between anterior and posterior commissure with a tilt of 23.9° from plumb. As the whole brain was too large to be scanned in an appropriate quality, lower parts of cerebellum and medulla oblongata were left out so that the (frontal) cortex could be fully included. We applied T2* weighted echo planar sequence with a repetition time (TR) of 2 s and an Echo Time (TE) of 30 ms. The flip angle was 80 and the acquisition matrix contained 64 × 64 pixels. Following the functional scans, high resolution T1-weighted anatomical volumes (Magnetization Prepared Rapid Gradient Echo; MPRAGE) were acquired with a resolution of 1 mm. Slice thickness was 1mm, repetition time (TR) 2.3 s, echo time (TE) 4.38 ms and inversion time (TI) was 900 ms. The Flip Angle was 8. The Field of View was 256 × 256 × 160 slices/mm.

### MR Data Analysis

Functional imaging data were analyzed using AFNI software (Cox, 1996) (available at http://afni.nimh.nih.gov/afni). At the preprocessing stage functional scanning data were slice time corrected and motion corrected. Afterward, the functional data from each subject were co-registered and transformed into a standard space using the EPI template from SPM2.^2^ Finally, the data were spatially smoothed with a Gaussian kernel [full width at half maximum (FWHM) = 5 mm], and then rescaled to the percent signal change (calculated as estimated signal expressed as a percentage of the baseline signal).

At the subject level, the 3dDeconvolve AFNI module was used to carry out a multiple regression analysis with picture types of achievement versus control as regressors. Pictures functioned as events and corresponding regressors were convolved with a canonical BOLD response. The coefficients were produced for each condition from each participant, and the comparison of these coefficients yielded the contrast values. At the group level analysis, the whole-brain betas from the contrast model were regressed on implicit (i.e., OMT) versus explicit (i.e., PVQ) AM scores in separate regression analyses using the AFNI module 3DregAna. In preliminary analyses, we also conducted regression analyses for implicit and explicit AM controlling for explicit or implicit AM, respectively. Because implicit and explicit AM were uncorrelated (see section “Results”) and because the results of the controlled analyses did not substantially differ from the results of the non-controlled analyses, we only report on the more powerful (because of more degrees of freedom) non-controlled analyses. Clusters were thresholded at *p* = 0.01 (FWE-corrected) and at a minimum size of 20 voxels. Cluster locations are reported using Talairach coordinates.

**FIGURE 2:**
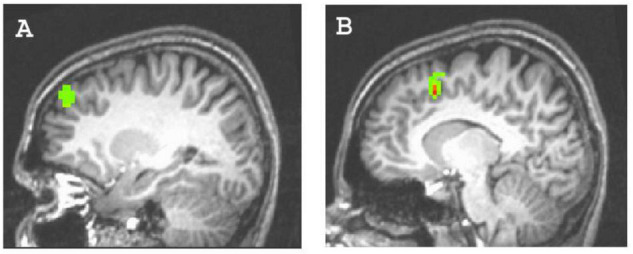
Clusters predicted by the explicit achievement motive. **(A)** Left dorsolateral prefrontal cortex activity (44 voxels; x,y,z = –24, 42, 30); **(B)** left anterior cingulate cortex activity (34 voxels; x,y,z = –9, 12, 39).

## Results

### Psychological Data

Participants rated achievement pictures (*M* = 7.40, *SD* = 1.21) as relating more strongly to achievement contents than control pictures (*M* = 2.45, *SD* = 1.43), *t*(12) = 11.64, *p* < 0.001. Although this was also the case for ratings about power contents (*M* = 3.40, *SD* = 1.66 vs. *M* = 1.05, *SD* = 1.52), *t*(12) = 5.67, *p* < 0.001, achievement pictures were rated excessively higher on achievement than on power, *t*(12) = 6.16, *p* < 0.001, which was expected. This suggests that the pictures stimulated the achievement rather than the power motive. In line with the literature (e.g., Brunstein and Heckhausen, 2018; McClelland et al., 1989), the correlation between implicit AM (*M* = 2.07, *SD* = 1.53) and explicit AM (*M* = 3.67, *SD* = 0.80) was not significant, *r* = 0.31, *p* = 0.26.

### Imaging Data

First, we investigated main effects of differences in brain activity between achievement versus control pictures by computing the corresponding contrast. We did not find significant differences. Investigating our primary research question, we regressed in two different models the contrast of achievement versus control pictures, either on the explicit or the implicit AM. We found that the explicit AM predicted activity in regions typically associated with planning and deliberate control, whereas the implicit AM predicted activity in regions typically associated with intuitive processing and action preparation, but not vice versa. Specifically (cf. Table 1), the explicit AM significantly predicted activations in the left ACC (BA 32), in two clusters of the right ACC (BA 32 and BA 24), and in the left DLPFC (BA 9). In addition, we found activation in the right fusiform gyrus (BA 20). By contrast, the implicit AM predicted activity in the left OFC (BA 47), in two clusters of the right dorsal premotor cortex (BA 6), and in bilateral occipital gyrus (BA 17). In addition, significant activity was predicted in the right DLPFC (BA 9) and reduced activity in the left parahippocampal gyrus (BA 37). We also conducted correlations of explicit and implicit motive scores with activity in regions of interest (ROI) typically involved in basic motivational processes, namely the nucleus accumbens (r_*e*_ = 0.10, r_*i*_ = 0.12), the amygdala (r_*e*_ = 0.05, r_*i*_ = 0.14), and the hypothalamus (r_*e*_ = 0.19, r_*i*_ = 0.13), with all results being far from significant, all *p*’s > 0.20.

**TABLE 1:** Activation clusters of achievement greater control pictures predicted by implicit versus explicit achievement motives.

X	Y	Z	Anatomical location	Brodmann area	Cluster size (vox)	*t*-value
***Explicit achievement motive***						
–24	42	30	L Dorsolateral Prefrontal Cortex*	9	44	4.15
18	12	27	R Anterior Cingulate Gyrus	24	37	5.63
–9	12	39	L Anterior Cingulate Gyrus*	32	34	7.46
45	–30	–18	R Fusiform Gyrus	20	31	7.78
15	21	39	R Anterior Cingulate Gyrus	32	24	7.56
***Implicit achievement motive***						
24	–81	15	R Middle Occipital Gyrus	17	92	6.34
–24	–45	6	L Parahippocampal Gyrus*	37	74	–5.60
–18	–87	12	L Cuneus	17	52	5.14
33	–3	48	R Premotor Cortex*	6	46	5.19
–24	15	–9	L Orbitofrontal Cortex/Insula*	11/13/47	31	5.92
51	9	27	R Dorsolateral Prefrontal Cortex*	9	27	5.32
30	–12	57	R Premotor Cortex	6	20	5.36

*Significant clusters thresholded at p = 0.005. Talairach coordinates.*

**FIGURE 3:**
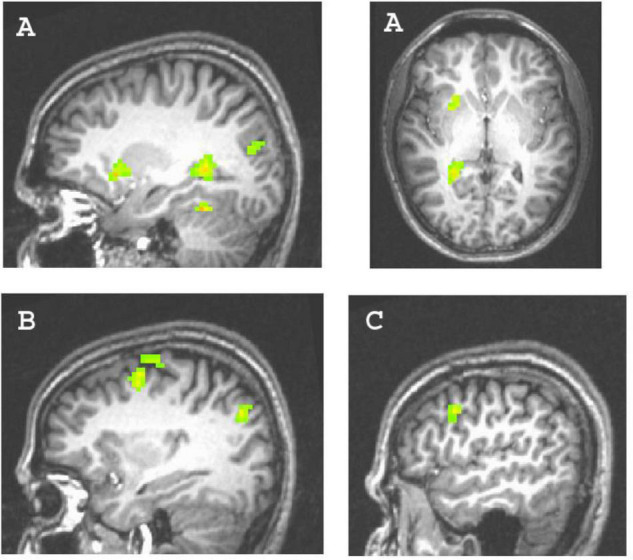
Clusters predicted by the explicit achievement motive. **(A)** Left parahippocampal gyrus activity (74 voxels; x,y,z = –24, –45, 6) and left orbitofrontal cortex activity (31 voxels; x,y,z = –24, –15, 9); **(B)** dorsal premotor cortex activity (46 voxels; x,y,z = 33, –3, 48); **(C)** right dorsolateral prefrontal cortex activity (27 voxels; x,y,z = 51, 9, 27).

*Clusters depicted in Figures 2, 3.

## Discussion

Based on McClelland’s (1985) theory of motives, explicit motives should predict brain activity in areas associated with controlled and planned behavior whereas implicit motives should predict activity in areas associated with areas intuitively guiding behavior on the basis of integrated values and emotion (see also Lieberman et al., 2002). Our findings were largely in line with this notion and will be discussed below. They are also in line with notions about brain areas underlying implicit and explicit attitudes (Lieberman et al., 2002) and speak to the validity of the Operant Motives Test as a procedure for the assessment of implicit motives.

Congruent with our hypothesis, the implicit AM predicted activity in the OFC (BA 11/47) and the insula (BA 13)—areas that represent the implicit value of potential rewards in cued responses (Craig, 2002; Gottfried et al., 2003; Kringelbach, 2005), and accordingly, is active when processing emotionally arousing pictures (Phan et al., 2004, for a review). Bradley et al. (2003) suggest that increased OFC activity reflects motivated attention in a way that “motivational engagement directs attention and facilitates perceptual processing” (p. 369). Accordingly, the OFC finding is also in line with the assumption that the implicit AM is closely related to pictorial processing. It may be speculated that individuals with a high implicit AM show increased activity in OFC because of increased automatic or intuitive motivational engagement and interest in achievement-related scenes.

The present finding is also compatible with the OFC’s and insula’s role in decision-making (Bechara et al., 2000; Padoa-Schioppa and Assad, 2006). Specifically, when individuals are able to recruit their emotional preferences (such as for engaging in achievement-related situations) and related neural areas (such as the OFC/insula), they are better able to make satisfying decisions. In fact, the intersection of BA 47 and BA 11 of the OFC, as found here, seems specifically related to the representation of personal goal values as compared to decision-making (Hare et al., 2008). As to parahippocampal activation, its inverse relationship with the implicit AM may suggest that individuals with a high implicit AM have recalled the meaning of the pictures better than those with a low implicit AM. Better memory for experiences congruent with the related implicit motive have been reported in previous studies (Bender and Woike, 2010, for a review).

Our findings on DLPFC activity are in line with our hypotheses as well. The DLPFC, right or left, is typically involved in planning and working memory (Fuster, 1995; Goldman-Rakic, 1995) as well as in self-control (Beauregard et al., 2001). Specifically, we found that the implicit AM predicted activity in the right DLPFC, whereas the explicit AM predicted activity in the left DLPFC. The left DLPFC has been found to be preferentially involved in the analysis of propositional aspects of a plan (Grafman et al., 2005), in representing the hierarchical sequence of subgoals to achieve a superordinate goal (Kaller et al., 2011), and in processing verbal rather than pictorial information (Devlin et al., 2003; Floel et al., 2004; Köhler et al., 2004). By contrast, the right DLPFC is preferentially involved in temporal and dynamic aspects of planning, movement monitoring, and integrating relevant information into action sequences (Grafman et al., 2005; Kaller et al., 2011), as well as in processing pictorial information in general (Floel et al., 2004). This is compatible with our hypothesis that motive-relevant stimuli (e.g., pictures, as used here) should induce a mode of propositional goal processing and memory rehearsal of verbal task instructions in individuals with high levels of explicit AM. By contrast, motive-relevant stimuli should activate pictorial goal representations and momentary performance-related representations constitutive of the experiencing of motivational flow in individuals with a high implicit AM (Csikszentmihalyi, 1990; Schultheiss and Brunstein, 2010). As such, individuals with a high implicit rather than high explicit AM might more readily translate goal-relevant pictorial information into propositional information. This could be a reason of increased intrinsic achievement motivation in individuals with a high implicit AM.

Left DLPC activation in explicit AM is also in line with research demonstrating that actions based on implemented intentions are more effortless and easier to be initiated than deliberatively intended actions (Gollwitzer, 1999; Brandstätter et al., 2001). Moreover, motivation and effort increases with the number of subgoals necessary to obtain a superordinate goal (Klapp and Erwin, 1976; see Lewin, 1936, for this theoretical consideration), which might be associated with explicit more so than with implicit AM.

In line with our hypotheses we found that the explicit AM predicted activity in BA 24 and BA 32 of the dorsal ACC. The ACC is typically implicated in controlled processing such as task monitoring, error detection, attentional control or conflict resolution (Botvinick et al., 2001; Shackman et al., 2011). In concert with the DLPFC (see above), the ACC forms a cognitive control network, which is typically implicated in a number of experimental cognitive tasks (e.g., Cole and Schneider, 2007; Dosenbach et al., 2008). In fact, BAs 32 and 24 of the ACC are jointly activated with BA 9 of the DLPFC in the Stroop paradigm when participants have to name but not to read the ink of a color word, with the former areas being involved in task monitoring and the latter being involved in task preparation (MacDonald et al., 2000). This is very much in line with the notion that explicit AM predicts consciously intended and controlled behavioral responses (McClelland et al., 1989) and is compatible with the notion that individuals with high levels of explicit AM rely on effortful cognitive self-control when initiating demanding tasks (e.g., Kuhl and Fuhrmann, 1998; Kehr, 2004).

The finding of right premotor (BA 6) activity for the implicit AM was unpredicted. The dorsal part of BA 6 (z > 45), as found here, is a central part of the human mirror system and plays a major role in action imitation as a specific manifestation of intuitive behavior (e.g., Molenberghs et al., 2009). As such, this finding is in line with the notion of implementation intentions discussed above (Gollwitzer, 1999). Specifically, it might be speculated that individuals with a high implicit AM might show mirror neuron system activity while putting themselves in the place of the depicted individual, and might thus more readily imitate behavior on a mental level. Such a process might constitute a neural underpinning of effortless and easy task implementation in everyday situations for individuals with a high AM (Quirin et al., 2021). Mirror neuron system activation might even cooperate with the right DLPFC in facilitating implementation intentions.

We did not find activation of areas typically involved in elementary motivational processes such as the nucleus accumbens, the amygdala, or the hypothalamus. Achievement motivation (and motivation in general) but not the motive is typically considered to result from both incentive value and expectancy (to attain the incentive goal) (Berridge, 2012; Beckmann and Heckhausen, 2018). In the present study, however, the presentation of pictures did not render participants to expect the attainment of a goal. Neither were the pictures of high incentive value. Accordingly, the relative absence of activation of the mentioned regions is likely the result of the study design we chose to investigate implicit and explicit AMs. Accordingly, areas found active are less likely to reflect correlates of manifest achievement motivation. Rather, they are likely to represent neural correlates of AMs in terms of a preference (sense of value) for achievement-related, challenging tasks.

Although the present findings may not immediately refer to motivation (but to motive stimulation), they may explain brain mechanisms determining how individuals can accomplish demanding tasks in everyday life even in the absence of motivation. Typically, when implicit AM is low, high levels of self-control can buffer the lack of basic motivational resources such that taxing tasks can be accomplished nevertheless (Kuhl and Fuhrmann, 1998; Kehr, 2004). Accordingly, the present findings suggest that a neural network including ACC and left DLPFC may be crucial in supporting this function. High levels of self-control as potentially represented by the ability to recruit these neural structures may thus help individuals manage everyday life requirements or job-related demands and to buffer against procrastination, especially when task-inherent incentives are low or even aversive. This is in line with previous research (Langan-Fox et al., 2009) showing that high levels of self-control buffers against reductions of well-being and physical health and other symptoms that can result from a high explicit AM when it is not backed up by the implicit AM (e.g., Kehr, 2004; Baumann et al., 2005).

Previous research has documented the validity of implicit motive measures in general (Schultheiss and Brunstein, 2010), and the operant motive test in particular. The present findings extend the validity of implicit motive measures within a neuroscientific context. Lending support to the dual-systems theory of motives (McClelland et al., 1989), the present findings endorse joint assessment of explicit and implicit motives for the analysis of motivational processes, not only in scientific research but also interventional contexts such as psychotherapy, organizational psychology, and school psychology (Alsleben and Kuhl, 2010).

The AM, implicit or explicit, is not an entirely homogeneous construct. Rather, subclasses of the AM have been distinguished in the literature, such as hope fors success and fear of failure (e.g., Brunstein and Heckhausen, 2018), or even more finegrained levels (Baumann and Kuhl, 2020). While the present research constitutes pioneered neural correlates of implicit versus explicit AMs, future research may investigate different subtypes of the AM using more finegrained motive scales in a larger sample of participants. At least three limitations of the present study should be mentioned. First, we did not find significant main effects when contrasting achievement-related with control pictures. It should be noted that main effect findings would not have been able to distinguish between neural correlates of implicit and explicit AMs, which was key to the present research. Nevertheless, finding main effects would have been desirable and different reasons for their absence or a combination of them may be plausible: The drawings used here might not have been strong enough to arouse achievement motivation because of low self-relevance of the pictures or habituation effects due to repeated presentation. Moreover, according to common motive theories, motivation is not only provided by incentive intensity (and a strong related motive) but also by the expectancy to attain the incentive stimulus or goal. Because we presented pictures of challenging tasks but did not provide the opportunity (and thus the expectancy) to attain a specific task goal, achievement motivation and corresponding activation likely did not become manifest. Future research may therefore use stimuli of more intense incentive and expectancy values (e.g., increasing self-relevance, using original rather than repeated stimuli only).

Second, the sample size is relatively small regarding contemporary conventions. Accordingly, the present study should be treated as a pilot study for future investigations of the neural correlates of implicit versus explicit AMs. Nevertheless, the small sample size was (at least partially) compensated by a high duration of scanning time of about 16 min for the experimental condition. We also conducted a *post hoc* statistical power analysis using G-power software (parameter set to 0.8, alpha error probability set to 0.05, *t*-value averaged across significant areas = 4.15), which resulted in a number of *N* = 36 participants—a finding that future fMRI studies on the AM using a similar study design may want to build upon.

Third, the present study did not provide any behavioral variables that could be used to validate the interpretation of the activations found. As such, despite the strong accordance of the majority of findings with the dual systems theory of implicit versus explicit motives, it cannot be excluded that the activations found may be interpreted differently. Therefore, future studies may use behavioral measures to validate the present interpretations, for example, in the context of a goal pursuit task that induces achievement motivation and that participants can succeed or fail at.

In conclusion, the present study is the first to contrast neural correlates of implicit and explicit motives drawing on the AM as a representative example. Our findings are in line with dual-systems theory of motives, suggesting that explicit motives predict effortful, planned actions, whereas implicit motives predict effortless, spontaneous actions (McClelland, 1985; McClelland et al., 1989). Although this pilot study is not without limitations, it sheds first light on the neural mechanisms potentially accompanying these two types of motives and may serve as a foundation for an entire research program in the area of motives.

## Data Availability Statement

The raw data supporting the conclusions of this article will be made available by the authors.

## Ethics Statement

The studies involving human participants were reviewed and approved by University of Osnabrück, Germany. The participants provided their written informed consent to participate in this study.

## Author Contributions

MQ, AL, and JK conceptualized the study. AL and MQ implemented the procedure, conducted the study, analyzed the data, and drafted the manuscript. EK supervised all questions concerning fMRI and guided the MR assessments. All authors contributed to the article and approved the submitted version.

## Conflict of Interest

The authors declare that the research was conducted in the absence of any commercial or financial relationships that could be construed as a potential conflict of interest.

## Publisher’s Note

All claims expressed in this article are solely those of the authors and do not necessarily represent those of their affiliated organizations, or those of the publisher, the editors and the reviewers. Any product that may be evaluated in this article, or claim that may be made by its manufacturer, is not guaranteed or endorsed by the publisher.

## Footnotes

1 Because the quality and intensity of mental processes might depend on picture duration, we explored two different durations. However, because the results were similar but non-significant, we aggregated the two conditions.

2
www.fil.ion.ucl.ac.uk/spm/

## References

[B1] Ainslie G. (2021). Willpower with and without effort. *Behav. Brain Sci.* 44:e30. 10.1017/S0140525X20000357 32843105 PMC9280284

[B2] Alsleben P. Kuhl J. (2010). “Touching a person’s essence: Using implicit motives as personal resources in counseling,” in *Handbook of motivational counseling: Motivating People for Change*, 2 Edn, eds Cox W. M. Klinger E. (Sussex, UK: Wiley), 109–131.

[B3] Apers C. Lang J. W. B. Derous E. (2019). Who earns more? Explicit traits, implicit motives and income growth trajectories. *J. Vocat. Behav.* 110 214–228.

[B4] Atkinson J. W. McClelland D. C. (1948). The projective expression of needs. II. The effects of different intensities of the hunger drive on thematic apperception. *J. Exp. Psychol.* 28 643–658. 10.1037/h0061442 18893180

[B5] Baumann N. Kaschel R. Kuhl J. (2005). Striving for unwanted goals: stress-dependent discrepancies between explicit and implicit achievement motives reduce subjective well-being and increase psychosomatic symptoms. *J. Pers. Soc. Psychol.* 89 781–799. 10.1037/0022-3514.89.5.781 16351368

[B6] Baumann N. Kuhl J. (2020). Nurturing your self: measuring and changing how people strive for what they need. *J. Posit. Psychol.* 16 726–737. 10.1080/17439760.2020.1805503

[B7] Beauregard M. Lévesque J. Bourgouin P. (2001). Neural correlates of conscious self-regulation of emotion. *J. Neurosci.* 21:RC165. 10.1523/JNEUROSCI.21-18-j0001.2001 PMC6763007 11549754

[B8] Bechara A. Damasio H. Damasio A. R. (2000). Emotion, decision making and the orbitofrontal cortex. *Cereb. Cortex* 10 295–307. 10.1093/cercor/10.3.295 10731224

[B9] Beckmann J. Heckhausen H. (2018). “Motivation as a function of expectancy and incentive,” in *Motivation and Action*, eds Heckhausen J. Heckhausen H. (London, UK: Cambridge University Press), 163–220.

[B10] Bender M. Woike B. A. (2010). “Learning and memory correlates of implicit motives,” in *Implicit Motives*, eds Schultheiss O. C. Brunstein J. C. (New York, NY: Oxford University Press), 211–244.

[B11] Berridge K. C. (2012). From prediction error to incentive salience: mesolimbic computation of reward motivation. *Eur. J. Neurosci.* 35 1124–1143. 10.1111/j.1460-9568.2012.07990.x 22487042 PMC3325516

[B12] Botvinick M. M. Braver T. S. Barch D. M. Carter C. S. Cohen J. D. (2001). Conflict monitoring and cognitive control. *Psychol. Rev.* 108 624–652. 11488380 10.1037/0033-295x.108.3.624

[B13] Bradley M. M. Sabatinelli D. Lang P. J. Fitzsimmons J. R. King W. Desai P. (2003). Activation of the visual cortex in motivated attention. *Behav. Neurosci.* 117 369–380. 10.1037/0735-7044.117.2.369 12708533

[B14] Brandstätter V. Lengfelder A. Gollwitzer P. M. (2001). Implementation intentions and efficient action initiation. *J. Pers. Soc. Psychol.* 81 946–960. 10.1037//0022-3514.81.5.946 11708569

[B15] Brunstein J. C. (2001). Persönliche Ziele und Handlungs- versus Lageorientierung: wer bindet sich an realistische und bedürfniskongruente Ziele? [Personal goals and action versus state orientation: who builds a commitment to realistic and need-congruent goals?]. *Z. für Differentielle und Diagnostische Psychol.* 22 1–12. 10.1024//0170-1789.22.1.1

[B16] Brunstein J. C. Heckhausen H. (2018). “Achievement Motivation,” in *Motivation and Action*, eds Heckhausen J. Heckhausen H. (London, UK: Cambridge University Press), 221–304.

[B17] Brunstein J. C. Schultheiss O. C. Grassmann R. (1998). Personal goals and emotional well-being: the moderating role of motive dispositions. *J. Pers. Soc. Psychol.* 75 494–508. 10.1037//0022-3514.75.2.494 9731321

[B18] Cole M. W. Schneider W. (2007). The cognitive control network: integrated cortical regions with dissociable functions. *Neuroimage* 37 343–360. 10.1016/j.neuroimage.2007.03.071 17553704

[B19] Cox R. W. (1996). AFNI – Software for Analysis and Visualization of Functional Magnetic Resonance Neuroimages. *Comput. Biomed. Res.* 29 162–173. 8812068 10.1006/cbmr.1996.0014

[B20] Craig A. D. (2002). How do you feel? interoception: the sense of the physiological condition of the body. *Nat. Rev. Neurosci.* 3, 655–666. 10.1038/nrn894 12154366

[B21] Csikszentmihalyi M. (1990). *Flow: The Psychology of Optimal Experience.* New York, NY: Harper & Row.

[B22] Deci E. L. Ryan R. M. (2008). Self-determination theory: a macrotheory of human motivation, development, and health. *Can. Psychol.* 49 182–185. 10.1037/a0012801

[B23] Devlin J. T. Matthews P. M. Rushworth M. F. (2003). Semantic processing in the left inferior prefrontal cortex: a combined functional magnetic resonance imaging and transcranial magnetic stimulation study. *J. Cogn. Neurosci.* 15 71–84. 10.1162/089892903321107837 12590844

[B24] Dosenbach N. U. Fair D. A. Cohen A. L. Schlaggar B. L. Petersen S. E. (2008). A dual-networks architecture of top-down control. *Trends Cogn. Sci.* 12 99–105. 10.1016/j.tics.2008.01.001 18262825 PMC3632449

[B25] Elliot A. J. Thrash T. M. (2001). Achievement goals and the hierarchical model of achievement motivation. *Educ. Psychol. Rev.* 13 139–156.

[B26] Floel A. Poeppel D. Buffalo E. A. Braun A. Wu C. W.-H. Seo H.-J. (2004). Prefrontal Cortex Asymmetry for Memory Encoding of Words and Abstract Shapes. *Cereb. Cortex* 14 404–409. 10.1093/cercor/bhh002 15028644

[B27] Fuster J. M. (1995). *Memory in the Cerebral Cortex.* Cambridge, MA: MIT Press.

[B28] Goldman-Rakic P. S. (1995). Architecture of the prefrontal cortex and the central executive. *Ann. N. Y. Acad. Sci.* 769 71–83. 10.1111/j.1749-6632.1995.tb38132.x 8595045

[B29] Gollwitzer P. M. (1999). Implementation intentions: strong effects of simple plans. *Am. Psychol.* 54 493–503.

[B30] Gottfried J. A. O’Doherty J. Dolan R. J. (2003). Encoding predictive reward value in human amygdala and orbitofrontal cortex. *Science* 301 1104–1107. 10.1126/science.1087919 12934011

[B31] Grafman J. Spector L. Rattermann M. J. (2005). “Planning and the brain,” in *The Cognitive Psychology of Planning*, eds Morris R. Ward G. (Hove, UK: Psychology Press), 181–198.

[B32] Hare T. A. O’Doherty J. Camerer C. F. Schultz W. Range A. (2008). Dissociating the role of the orbitofrontal cortex and the striatum in the computation of goal values and prediction errors. *J. Neurosci.* 28 5623–5630. 10.1523/JNEUROSCI.1309-08.2008 18509023 PMC6670807

[B33] Hofer J. Busch H. Chasiotis A. Kiessling F. (2006a). Motive congruence and interpersonal identity status. *J. Pers.* 74 511–542. 10.1111/j.1467-6494.2006.00383.x 16529585

[B34] Hofer J. Chasiotis A. Campos D. (2006b). Congruence between social values and implicit motives: effects on life satisfaction across three cultures. *Eur. J. Pers.* 20 305–324.

[B35] Kaller C. P. Rahm B. Spreer J. Weiler C. Unterrainer J. M. (2011). Dissociable contributions of left and right dorsolateral prefrontal cortex in planning. *Cereb. Cortex* 21 307–317. 10.1093/cercor/bhq096 20522540

[B36] Kehr H. M. (2004). Implicit/explicit motive discrepancies and volitional depletion among managers. *Pers. Soc. Psychol. Bull.* 30 315–327. 10.1177/0146167203256967 15030623

[B37] Klapp S. T. Erwin C. I. (1976). Relation between programming time and duration of the response being programmed. *J. Exp. Psychol.* 2 591–598. 10.1037//0096-1523.2.4.591 1011008

[B38] Köhler S. Paus T. Buckner R. L. Milner B. (2004). Effects of left inferior prefrontal stimulation on episodic memory formation: a two-stage fMRI-rTMS study. *J. Cogn. Neurosci.* 16 178–188. 10.1162/089892904322984490 15068590

[B39] Kringelbach M. L. (2005). The human orbitofrontal cortex: linking reward to hedonic experience. *Nat. Rev. Neurosci.* 6 691–702. 10.1038/nrn1747 16136173

[B40] Kuhl J. (2000). “A functional-design approach to motivation and volition: The dynamics of personality systems interactions,” in *Self-regulation: Directions and challenges for future research*, eds Boekaerts M. Pintrich P. R. Zeidner M. (New York, NY: Academic Press), 111–169.

[B41] Kuhl J. Fuhrmann A. (1998). “Decomposing self-regulation and self-control: The volitional components checklist,” in *Life span perspectives on motivation and control*, eds Heckhausen J. Dweck C. (Mahwah, NJ: Erlbaum), 15–49.

[B42] Kuhl J. Kazén M. (2008). Motivation, affect, and hemispheric asymmetry: power versus intimacy. *J. Pers. Soc. Psychol.* 95 456–469. 10.1037/0022-3514.95.2.456 18665713

[B43] Kuhl J. Scheffer D. Eichstaedt J. (2003). “Der Operante Motiv-Test (OMT): Ein neuer Ansatz zur Messung impliziter Motive,” in *Diagnostik von Motivation und Selbstkonzept*, eds Rheinberg F. Stiensmeier-Pelster J. (Göttingen: Hogrefe), 129–149.

[B44] Langan-Fox J. Sankey M. J. Canty J. M. (2009). Incongruence between implicit and self-attributed achievement motives and psychological well-being: the moderating role of self-directedness, self-disclosure and locus of control. *Pers. Individ. Differ.* 47 99–104.

[B45] Langens T. (2003). Daydreaming mediates between goal commitment and goal attainment in individuals high in achievement motivation. *Imagin. Cogn. Pers.* 22 103–115.

[B46] Langens T. A. (2007). Congruence between implicit and explicit motives and emotional wellbeing: the moderating role of activity inhibition. *Motiv. Emot.* 31 49–59.

[B47] Lewin K. (1936). *Principles of Topological Psychology.* New York, NY: McGraw-Hill.

[B48] Lieberman M. D. Gaunt R. Gilbert D. T. Trope Y. (2002). “Reflexion and reflection: A social cognitive neuroscience approach to attributional inference,” in *Advances in Experimental Social Psychology*, ed. Zanna M. P. (New York, NY: Academic Press), 199–249. 10.1016/j.concog.2007.12.004

[B49] MacDonald A. W. Cohen J. D. Stenger V. A. Carter C. S. (2000). Dissociating the role of the dorsolateral prefrontal and anterior cingulate cortex in cognitive control. *Science* 288 1835–1838. 10.1126/science.288.5472.1835 10846167

[B50] McClelland D. C. (1985). *Human Motivation.* Glenview, IL: Scott, Foresman.

[B51] McClelland D. C. (1991). *The Personal Value Questionnaire.* Boston, MA: McBer & Company.

[B52] McClelland D. C. Atkinson J. W. Clark R. A. Lowell E. L. (1953). *The Achievement Motive.* New York, NY: Appleton-Century-Crofts.

[B53] McClelland D. C. Boyatzis R. E. (1982). Leadership motive pattern and long-term success in management. *J. Appl. Psychol.* 67 737–743.

[B54] McClelland D. C. Franz C. E. (1992). Motivational and other sources of work accomplishment in mid-life: a longitudinal study. *J. Pers.* 60 679–707.

[B55] McClelland D. C. Koestner R. Weinberger J. (1989). How do self-attributed and implicit motives differ? *Psychol. Rev.* 96 690–702.

[B56] Molenberghs P. Cunnington R. Mattingley J. B. (2009). Is the mirror neuron system involved in imitation? A short review and meta-analysis. *Neurosci. Biobehav. Rev.* 33 975–980. 10.1016/j.neubiorev.2009.03.010 19580913

[B57] Morgan C. D. Murray H. A. (1935). A method for investigating fantasies: the thematic appercepation test. *Arch. Neurol. Psychiatry* 34, 289–306. 10.1001/archneurpsyc.1935.02250200049005

[B58] Murray H. A. (1938). *Explorations in Personality.* New York, NY: Oxford University Press.

[B59] Murray H. A. (1943). *Thematic Apperception Test Manual.* Cambridge, MA: Harvard University Press.

[B60] Padoa-Schioppa C. Assad J. A. (2006). Neurons in the orbitofrontal cortex encode economic value. *Nature* 441 223–226. 10.1038/nature04676 16633341 PMC2630027

[B61] Phan K. L. Wager T. D. Taylor S. F. Liberzon I. (2004). Functional neuroimaging studies of human emotions. *CNS Spectr.* 9 258–266. 15048050 10.1017/s1092852900009196

[B62] Quirin M. Beckenkamp M. Kuhl J. (2009). Giving or taking: the role of dispositional power motivation and positive affect in profit maximization. *Mind Soc.* 8 109–126.

[B63] Quirin M. Meyer F. Heise N. Kuhl J. Küstermann E. Strüber D. (2013). Neural correlates of social motives: an fMRI study on power versus affiliation. *Int. J. Psychophysiol.* 88 289–295. 10.1016/j.ijpsycho.2012.07.003 22841755

[B64] Quirin M. Jais M. Di Domenico S. I. Kuhl J. Ryan R. M. (2021). Effortless willpower? the integrative self and self-determined goal pursuit. *Front. Psychol*. 12. 10.3389/fpsyg.2021.653458 33815234 PMC8012899

[B65] Runge J. M. Lang J. W. B. Engeser S. Schüler J. den Hartog S. C. Zettler I. (2016). Modeling motive activation in the Operant Motive Test: a psychometric analysis using dynamic Thurstonian item response theory. *Motiv. Sci.* 2 268–286.

[B66] Scheffer D. Kuhl J. Eichstaedt J. (2003). “Der Operante Motiv-Test (OMT): Inhaltsklassen, Auswertung, psychometrische Kennwerte und Validierung,” in *Diagnostik von Motivation und Selbstkonzept*, eds Rheinberg F. Stiensmeier-Pelster J. (Göttingen: Hogrefe), 151–168.

[B67] Schneider W. Eschman A. Zuccolotto A. (2002). *E-Prime User’s Guide.* Pittsburgh, PA: Psychology Software Tools Inc.

[B68] Schüler J. Sheldon K. Fröhlich S. (2010). Implicit need for achievement moderates the relationship between felt competence and subsequent motivation. *J. Res. Pers.* 44 1–12.

[B69] Schultheiss O. C. Brunstein J. C. (1999). Goal imagery: bridging the gap between implicit motives and explicit goals. *J. Pers.* 67 1–38.

[B70] Schultheiss O. C. Brunstein J. C. (2001). Assessment of implicit motives with a research version of the TAT: picture profiles, gender differences, and relations to other personality measures. *J. Pers. Assess.* 77 71–86. 10.1207/S15327752JPA7701_05 11562105

[B71] Schultheiss O. C. Brunstein J. C. (2010). “Introduction,” in *Implicit Motives*, eds Schultheiss O. C. Brunstein J. C. (New York, NY: Oxford University Press).

[B72] Schultheiss O. C. Wirth M. M. (2008). “Biopsychological aspects of motivation,” in *Motivation and Action*, eds Heckhausen J. Heckhausen H. (London, UK: Cambridge University Press). 10.1186/2193-1801-2-246

[B73] Schultheiss O. C. Wirth M. M. Waugh C. E. Stanton S. J. Meier E. Reuter-Lorenz P. (2008). Exploring the motivational brain: effects of implicit power motivation on brain activation in response to facial expressions of emotion. *Soc. Cogn. Affect. Neurosci.* 3 333–343. 10.1093/scan/nsn030 19015083 PMC2607053

[B74] Shackman A. J. Salomons T. V. Slagter H. A. Fox A. S. Winter J. J. Davidson R. J. (2011). The integration of negative affect, pain, and cognitive control in the cingulate cortex. *Nat. Rev. Neurosci.* 12 154–167. 10.1038/nrn2994 21331082 PMC3044650

[B75] Spangler W. D. (1992). Validity of questionnaire and TAT measures of need for achievement: two meta-analyses. *Psychol. Bull.* 112 140–154.

[B76] Thrash T. M. Elliot A. J. Schultheiss O. C. (2007). Methodological and dispositional predictors of congruence between implicit and explicit need for achievement. *Pers. Soc. Psychol. Bull.* 33 961–974. 10.1177/0146167207301018 17510281

[B77] Winter D. G. (1973). *The Power Motive.* New York, NY: The Free Press.

